# Supplementary material for: Tracing nerve fibers with volume electron microscopy to quantitatively analyze brain connectivity
Source: Commun Biol. 2024 Jul 1;7:796. doi: 10.1038/s42003-024-06491-0 (PMC11217374; doi:10.1038/s42003-024-06491-0)
Supplement: Supplementary file 2 — Supplementary Information [file 42003_2024_6491_MOESM2_ESM.pdf]

Supplementary Information

Tracing nerve fibers with volume electron microscopy to quantitatively analyze brain connectivity

Marta Turegano-Lopez, Felix de las Pozas, Andrea Santuy, Jose-Rodrigo Rodriguez, Javier DeFelipe, Angel Merchan-Perez

Region	Synapses/µm^3 (AS+SS)	AS/µm^3	SS/µm^3	Percent AS	Percent SS
SR	2.76	2.68	0.08	97.10%	2.90%
L1	1.80	1.60	0.20	88.89%	11.11%
L3	1.08	1.01	0.07	93.91%	6.09%

Supplementary Table S1. Densities and percentages of synapses. The densities of asymmetric and symmetric synapses (AS and SS, respectively) are given in synapses per cubic micron and in percentages, in the stratum radiatum (SR) and layers 1 and 3 of the somatosensory cortex (L1 and L3). Synapse density values have been corrected for tissue shrinkage.

Region	Synapses on dendritic spines (%)	Synapses on shafts of spiny dendrites (%)	Synapses on smooth dendrites (%)
SR	84.65%	8.08%	7.27%
L1	74.81%	23.43%	1.76%
L3	80.45%	17.72%	1.83%

Supplementary Table S2. Distribution of synapses on different dendritic targets. Percentages of synapses established on dendritic spines, the shaft of dendrites with spines (spiny dendrites) and on dendrites without spines (smooth dendrites).

	Dendrites with spines			Dendrites without spines
**Region**	**Total linear density of synapses (spines + shafts)**	**Linear density of synapses on spines**	**Linear density of synapses on shafts**	**Linear density of synapses**
SR	3.3349 ± 0.1654 (48)	3.0828 ± 0.1518 (48)	0.2521 ± 0.0294 (48)	1.8366 ± 0.6780 (6)
L1	1.7700 ± 0.1198 (66)	1.4136 ± 0.1134 (66)	0.3559 ± 0.0406 (66)	0.6510 ± 0.1935 (4)
L3	1.8773 ± 0.1768 (33)	1.5582 ± 0.1669 (33)	0.3191 ± 0.0493 (33)	0.6556 ± 0.1708 (2)

Supplementary Table S3. Linear densities of synapses established on dendrites. The linear densities of synapses are given as the number of synapses per micron of dendritic shaft ± standard error of the mean. In the case of dendrites with spines, the total linear density includes synapses on spines and synapses on the shaft. Data are given as synapses/µm ± standard error of the mean, with the number of dendrites in each group in brackets.

Region	AS on spines	AS on shafts	SS on spines	SS on shafts
SR	90.10% (1356)	7.44% (112)	0.73% (11)	1.73% (26)
L1	79.06% (759)	10.52% (101)	2.40% (23)	8.02% (77)
L3	87.85% (499)	5.99% (34)	2.46% (14)	3.70% (21)

Supplementary Table S4. Distribution of asymmetric and symmetric synapses (AS and SS, respectively) on spines and dendritic shafts in the stratum radiatum of the hippocampus (SR), and layers 1 and 3 of the somatosensory cortex (L1 and L3). Data are given as percentages, with the absolute numbers of synapses in brackets.

Region	Ratio AS on spines / AS on shafts	Ratio SS on spines / SS on shafts	Ratio AS on spines / SS on spines	Ratio AS on shafts / SS on shafts
SR	92.37% / 7.63% (1356/112)	29.73% / 70.27% (11/26)	99.20% / 0.80% (1356/11)	81.16% / 18.84% (112/26)
L1	88.26% / 11.74% (759/101)	23.00% / 77.00% (23/77)	97.06% / 2.94% (759/23)	56.74% / 43.26% (101/77)
L3	93.62% / 6.38% (499/34)	40.00% / 60.00% (14/21)	97.27% / 2.73% (499/14)	61.82% / 38.18% (34/21)

Supplementary Table S5. Ratios of asymmetric and symmetric synapses (AS and SS) established on spines and shafts in the stratum radiatum of the hippocampus (SR), and layers 1 and 3 of the somatosensory cortex (L1 and L3). The ratios are given as percentages, with the absolute numbers of synapses in brackets.

Region	**Excitatory axons Linear density of synapses** Synapses/µm ± sem (n)	**Inhibitory axons Linear density of synapses** Synapses/µm ± sem (n)
SR	0.3845 ± 0.0082 (479)	0.3294 ± 0.0455 (12)
L1	0.2293 ± 0.0066 (362)	0.3016 ± 0.0258 (33)
L3	0.2443 ± 0.0083 (252)	0.2589 ± 0.0414 (17)

Supplementary Table S6. Linear densities of synapses established by excitatory and inhibitory axons in the stratum radiatum (SR) and in layers 1 and 3 of the somatosensory cortex (L1 and L3). Data are given as Synapses/µm ± standard error of the mean, with the number of axons in each group in brackets.

Region	Excitatory axons with more than one synapse	Excitatory axons with more than one synapse on the same dendrite (%)	Inhibitory axons with more than one synapse	Inhibitory axons with more than one synapse on the same dendrite (%)	Dendrites with more than one synapse	Dendrites with more than one synapse from one axon (%)	Dendrites with more than one synapse from multiple axons (%)
SR	409	42 (10.27%)	9	3 (33.33%)	63	24 (38.10%)	8 (12.70%)
L1	211	17 (8.06%)	27	4 (14.81%)	82	17 (20.73%)	2 (2.44%)
L3	119	12 (10.08%)	10	2 (20.00%)	41	9 (21.95%)	2 (4.88%)

Supplementary Table S7. Axons and dendrites establishing multiple synapses. For excitatory and inhibitory axons, we indicate the number of axons examined that established more than one synapse and the proportions of these that established more than one synapse with the same dendrite. We also indicate the proportion of dendrites that establish multiple contacts with one axon and the proportions of dendrites that establish multiple synapses with more than one axon.

Region	Excitatory axons		Inhibitory axons	
	***En passant* synapses**	**Terminal synapses**	***En passant* synapses**	**Terminal synapses**
SR	95.44%	4.56%	97.30%	2.70%
L1	73.26%	26.74%	76.00%	24.00%
L3	80.49%	19.51%	85.71%	14.29%

Supplementary Table S8. *En passant* and terminal synapses. For excitatory and inhibitory axons, we indicate the percentages of synapses that were established *en passant*, and the percentages of synapses that were established by terminal boutons.
