# Supplementary material for: Tracing nerve fibers with volume electron microscopy to quantitatively analyze brain connectivity
Source: Commun Biol. 2024 Jul 1;7:796. doi: 10.1038/s42003-024-06491-0 (PMC11217374; doi:10.1038/s42003-024-06491-0)
Supplement: Supplementary file 3 — Description of Additional Supplementary Files [file 42003_2024_6491_MOESM3_ESM.pdf]

Description of Additional Supplementary Files

File name: Supplementary Movie 1

Description: To describe the connectivity of the tissue, we used a tracing tool available in Espina software. With this tool, the user can navigate the stack and trace any nerve fiber by following its trajectory with the mouse. Every tracing or “skeleton” is linked to its corresponding synaptic contacts, so the result is an intricate meshwork of axons and dendrites interconnected by a cloud of synaptic junctions. The software provides quantitative information regarding each skeleton, including shaft length, number of synapses and, in the case of dendrites, the number of dendritic spines.

File name: Supplementary Data 1.

Description: Raw data provided by Espina software regarding the skeletons of nerve fibers.
